# Gaps in Knowledge and the Need for Patient-Partners in Research Related to Physical Activity and Type 1 Diabetes: A Narrative Review

**DOI:** 10.3389/fendo.2019.00042

**Published:** 2019-02-06

**Authors:** Nika Klaprat, Andrea MacIntosh, Jonathan M. McGavock

**Affiliations:** ^1^Faculty of Kinesiology and Recreation Management, University of Manitoba, Winnipeg, MB, Canada; ^2^Diabetes Research Envisioned and Accomplished in Manitoba (DREAM) Theme, Children's Hospital Research Institute of Manitoba, Winnipeg, MB, Canada; ^3^Department of Pediatrics and Child Health, Faculty of Health Sciences, University of Manitoba, Winnipeg, MB, Canada; ^4^Diabetes Action Canada SPOR Network, Toronto, ON, Canada

**Keywords:** exercise, outcomes, epidemiology, clinical trials, cardiovascular disease, patient oriented research, priority setting, continuous glucose monitoring

## Abstract

Regular physical activity (PA) is a cornerstone in the management of complications associated with type 1 diabetes (T1D). Most national guidelines advocate for regular PA for persons living with T1D, however the evidence to support these recommendations has not be reviewed recently. Additionally, in an era of patient-centered care and patient oriented research, the role of patient partners in the area of PA and T1D interventions has never been explored. The purpose of this narrative review is to overcome these two gaps in the literature. Here we review selected epidemiological evidence and identify gaps in research that would add important information to guide practitioners and future guidelines. We also provide an overview of patient-oriented research projects co-developed with persons living with T1D. Significant gaps in the field include: (1) a lack of adequately powered prospective cohort studies using serial measures of PA and hard chronic disease end-points; (2) no multi-centered, highly powered, randomized controlled trials of PA, and long-term health outcomes; (3) little data on the role of new technologies to support PA-related behavior change, and (4) no trials that involved patients in the design and execution of PA-based clinical trials. This review provides a template for scientists and patient partners to develop future research priorities and agendas in the field.

## Introduction

### The Rationale for Additional Research in the Area of Physical Activity and Health Outcomes in Persons Living With Type 1 Diabetes

Rates of type 1 diabetes (T1D) have increased globally over the past 2 decades (1–4) and it currently is the most common endocrine condition in children and young adults (5). A diagnosis of T1D increases the risk of micro- and macro-vascular complications that reduce life expectancy by as much as 15 years (6–10). The increased morbidity associated with T1D is significantly reduced with improved blood glucose control (11, 12). In conjunction with carefully titrated insulin and dietary modifications, most national health guidelines strongly recommend participation in regular physical activity (PA) for achieving optimal health for people with T1D. Unfortunately, there is little experimental evidence available to guide recommendations and/or behavior modification for persons living with T1D, particularly children and adolescents (13). This review is distinct from recently published comprehensive reviews (14–19) that have addressed areas of weight management, glucose monitoring, managing glucose during exercise and the physiological responses to exercise. We suggest readers also read the comprehensive review by Riddell et al. (14) that emerged from a consensus conference hosted by the JRDF PEAK programme and the Exercise for Type One Diabetes (EXTOD) group as it provides an excellent overview on the strategies for safely adopting an active lifestyle for persons living with T1D. We also recommend a review by Codella et al. (19) as it provides a thorough overview of the physiological benefits of exercise for persons with T1D and the potential for new technologies in supporting a more active lifestyle. The purpose of this narrative review is to provide an updated overview of the highest quality evidence for what we know about PA for persons living with T1D, gaps in the literature that could guide future research programs and finally, explore the benefits of patient engagement and co-development of a research agenda for the next wave of research in the field. This narrative review will complement other recent reviews as it will include a larger scope of research designs to summarize and describe the current state of evidence and identify literature gaps. While the studies included were not selected using a systematic process, we attempted to restrict the discussion to those with the most representative samples, robust designs and clinically relevant end-points. Additionally, we will explore the role of patient partners and models for patient-centered priority setting in determining the next generation of clinical trials/studies for persons living with T1D, which has not been an included goal in previously published reviews in this area.

### The Benefits of Physical Activity on Health Outcomes in Persons Living With T1D

Evidence for most PA guidelines are derived largely from prospective observational cohort studies (20, 21). Recent studies exemplify the advantages of a prospective cohort design for studying the association between PA and health outcomes (22–24). With adequately powered, representative samples, PA can be quantified across various domains (intensity, duration, frequency, type, timing) and with sufficient follow-up, differences in major health-related outcomes, including mortality, can be compared across these domains. As is the current trend, these associations can be replicated in similar cohorts and confounders can be controlled for using state-of-the-art methods (25). As some of these landmark cohort studies include serial measures, it is also possible to assess the association between changes in behavior over time and health outcomes (26, 27); however, few studies have applied this approach to the study of PA and major chronic diseases. As changing and sustaining PA behavior is challenging, adequately powered randomized controlled trials with sufficient follow-up time to examine dose-specific effects are extremely rare. Therefore, prospective cohort studies provide the bulk of the evidence to date on the association between PA and health outcomes in the general population and persons living with T1D.

Observational studies clearly demonstrate that regular PA is associated with several health benefits for patients with T1D (28), including higher cardiorespiratory fitness, lower serum cholesterol, enhanced vascular health, more favorable body composition and higher quality of life (29–44). A large cross sectional study of 18,000 persons with T1D from clinics across Germany revealed that higher levels of PA were associated with lower HbA1c, BMI, risk of hypertension, and dyslipidemia (45). Collectively, these benefits may contribute to the lower risk of complications (46) and increased life expectancy seen in physically active persons with T1D (47, 48). The first prospective study to examine this question, the Pittsburgh Insulin Dependent Diabetes Mellitus Morbidity and Mortality Study (46, 48), relied on a cohort of 671 patients within a registry of ~2,000 patients with T1D. Activity was assessed using a standardized questionnaire during a clinical visit and patients were followed for up to 25 years for rates of macro- and micro-vascular complications. Among men, those that did not participate in sport-related activities were ~3-fold more likely (31 vs. 11%, *p* < 0.05) to develop macro-vascular disease, compared to those that did. In a follow-up study, the authors found that participation in competitive sports as an adolescent was associated with a reduced odds of nephropathy, neuropathy and macro-vascular disease, relative to those that did not participate in competitive sports during high school. This association was not observed in women and it is unclear to what extent the analyses controlled for confounders.

More recent studies of larger cohorts (~2,000–3,000) of persons living with T1D in Europe and Scandinavia suggest that the association between PA and long-term cardiovascular disease (CVD) risk is modest and may be modified by other lifestyle factors that cluster with activity levels, particularly smoking. The FinnDiane study has published several analyses of self-reported PA on all-cause and CVD-specific mortality (49, 50). FinnDiane enrolled ~5,000 adults with T1D into a cohort study in 1995, with 2,300–2,600 completing questionnaires on the type, duration and intensity of weekly PA. Early work revealed that participation in more frequent (>2 days/week vs. < 1 day) and intense activity (high degree of subjective shortness of breath and sweating vs. none) were both associated with a ~50% reduced risk of proteinuria and progression to chronic kidney disease (51). As of 2014, 270 participants in the cohort had died and ~300 lived with chronic kidney disease. In the two most recent analyses, mortality was 70–100% higher in the most sedentary sub-groups in the cohort compared to those the highest tertiles of total leisure time PA and intensity of PA (50, 51). Importantly however, the strength of these associations are significantly reduced when adjusted for key confounders, particularly smoking (50). Therefore, the precision of these estimates remains questionable. Similarly, a preliminary analysis of the Joslin 50 years Medalist cohort in which participants self-reported PA showed that those who reported exercising regularly experienced a 45% lower risk of mortality; however, similar to previous studies, these analyses were only adjusted for a handful of confounders (52). Incredibly, no systematic reviews with meta-analysis of prospective cohort studies of PA and health outcomes among persons with T1D has ever been published. Accordingly, the strength of these associations remains restricted to small cohorts followed for a limited number of years.

These associations provide promising evidence for a protective association of PA on CVD and mortality among persons living with T1D, however they are far from conclusive. The findings presented above could be significantly strengthened by (1) conducting a rigorous systematic review of observational studies; (2) conducting larger cohort studies or a meta-analysis of individual level data with longer follow-up using objective measures of PA; (3) creating cohort studies with serial measures of PA over time to determine the association between changes in PA and long term CVD risk and finally, (4) creating cohort studies that conduct more robust analyses using propensity score matching or instrumental variable analysis to rigorously control for measured and unmeasured confounding. Finally, as PA is a behavior that tracks from adolescence through to adulthood, there is a major gap in our understanding of PA behavior in adolescence and health outcomes in adulthood. As recently argued (53), there is a dire need for more robust epidemiological work in the area of PA and chronic disease risk among persons living with T1D.

## Clinical Trials of Physical Activity and Health Outcomes among persons living with Type 1 Diabetes

A series of systematic reviews of clinical trials of PA and health outcomes in persons with T1D were published in the past few years, by our group (54) and others (55, 56). The most recent systematic review published in May 2018 (55), included 15 randomized trials of aerobic-only exercise interventions lasting 12–26 weeks that included 596 participants. Of these, 11 reported changes in HbA1c, 7 changes in body weight and 6 changes in VO2peak. Fewer studies reported changes in blood pressure or serum lipid profiles. Meta-analysis of available clinical trials revealed that structured exercise had no effect on HbA1c (MD: −0.08%, 95% CI: −0.38, 0.22; *p* = 0.6). However, structured exercise lowered daily insulin requirements (MD: −0.23 IU/kg, 95% CI −0.37, −0.09; *p* = 0.002) and body weight MD: −2.20 kg, 95% CI: −3.79, −0.61; *p* = 0.007) and increased peak oxygen uptake (4.08 mLO_2_/kg/min, 95% CI 2.18, 5.98; *p* < 0.0001). The authors attempted to discern a dose effect of PA on health outcomes as others have done (57), however were significantly underpowered to compare interventions that achieved recommended weekly requirements for moderate to vigorous PA (>150 min), compared to those that did not. Similar effects were seen in a systematic review and meta-analysis of 10 randomized and 16 non-randomized trials of exercise interventions lasting 2–39 weeks among children and adolescents (56). A relatively small trial (*n* = 51) of adolescents with T1D (58), published 3 years after this review, found that 20 weeks of endurance training, delivered four times weekly for 60 min, reduced total daily insulin dose and improved body composition and cardiorespiratory fitness compared to controls. The program also elicited modest improvements in measures of left ventricular systolic function and submaximal total peripheral resistance compared to controls, without any change in glycemic control or systolic and diastolic blood pressure. Overall, there is insufficient clinical trial data available to determine if structured exercise reduces risk factors for CVD in persons with T1D. Accordingly, there is a need for highly powered randomized controlled trials with prolonged follow-up to determine the efficacy of structured exercise on CVD-related health outcomes among persons living with T1D.

## Behavioral Trials of Promoting PA among Persons Living with Type 1 Diabetes

Randomized trials of structured, supervised exercise provide insight into the physiological adaptations associated with increased PA. In contrast, behavioral trials provide insight into how best to motivate individuals to adopt a more active lifestyle (59). The most recent systematic review of randomized trials of behavior modification for persons with T1D was published in 2015 (60). The authors identified 27 trials with 2,351 participants with T1D that delivered an intervention to modify self-management behaviors, some of which included PA (60). The trials that measured HbA1c (*n* = 22) observed an overall effect size of 0.16 (95% CI: 0.0–0.3), suggesting modest improvements in glycemic control with behavioral interventions. This effect was nearly twice as large if the intervention was grounded in an established theoretical model (Cohen's d: 0.22 vs. 0.12, *p* < 0.001). An updated review with recently published trials has not been conducted and therefore the efficacy of behavioral-based interventions to increase PA among persons with T1D remains unclear.

In a recent behavioral pilot trial in the UK (61), 58 young adults (32 ± 11 years) were randomized to a motivational interviewing intervention to increase daily PA with near biweekly meetings with a registered nurse, or standard clinical care. Participants in the intervention arm were targeting 150 min of moderate to vigorous PA weekly initially, with a long-term goal of achieving 240 min of moderate to vigorous PA weekly (61). An analysis restricted to participants that completed all data collection procedures found that the intervention group increased their weekly moderate to vigorous PA by ~40 min and experienced a ~10% increase in peak oxygen uptake. The control group decreased their weekly moderate to vigorous PA and experienced a ~10% decline in peak oxygen uptake. Patients in the intervention arm also experienced improvements in insulin sensitivity and reductions in total daily insulin relative to those in the control arm. This pilot trial suggests that motivational interviewing may be effective for increasing weekly PA among persons with T1D and that these changes may elicit benefits in the determinants of metabolic control, however larger trials with more prolonged follow-up are needed to confirm these results.

An excellent example of a clinically relevant behavioral intervention to support increased PA was recently published by the FLEX study group (62). An 18 months intervention that relied on motivational intervention and problem solving skills significantly improved quality of life and motivation for self-care among adolescents with T1D. Flexibility in program delivery and strategies to overcome barriers were core elements for eliciting behavior change. As the authors did not quantify PA behavior, it would be interesting to determine if a similar approach, focused on barriers to being active, would elicit similar effects, particularly among adults living with T1D.

The major take-away from these systematic reviews and recently published trials is that there is a glaring lack of adequately powered clinical trials of PA on health-related outcomes in persons living with T1D (Summarized in Table 1). Furthermore, among those published to date, few have reported outcomes beyond glycemic control and bodyweight. Therefore, there is a major gap in our understanding of the role, dose and long-term effectiveness of PA on clinically-relevant health outcomes (cardio-renal risk, mental health, quality of life, diabetes self-management) among persons with T1D. Adequately powered randomized trials with an extended long-term follow-up, similar to the Diabetes Prevention Program (63), The Finnish Diabetes Prevention Study (64) and the Look-AHEAD trial (65) are needed to provide clear evidence for the long-term health benefits of increasing PA among persons living with T1D.

**Table 1:** Knowledge and gaps in epidemiological research of physical activity for persons living with type 1 diabetes.

**Type of study**	**Physical activity knowledge**	**Gaps**
Prospective Cohort Studies	Lower lipid profiles Lower BP Reduced risk of CVD	Small sample sizes Lack of triangulation of data Poor control of confounding
	Reduced mortality	Lack of objective measures of PA
Clinical Trials	No effect on HbA1c Lower daily insulin requirement Weight loss Increased VO_2_peak	Efficacy for lowering BP Efficacy for improving lipid profiles
Behavioral trials	Improved self-management Reduced HbA1c Increased short-term PA	Optimal theoretical model for long term adherence to PA Optimal delivery model for increasing PA

*BP, blood pressure; CVD, cardiovascular disease; HbA1c, glycated hemoglobin; PA, physical activity; VO_2_, oxygen uptake*.

## Physical Activity and Hypoglycemia in Persons with Type 1 Diabetes

Hypoglycemia is the most common and life-threatening acute complication for persons living with T1D. It is also a key barrier to achieving optimal glycaemic control (66, 67). In the seminal Diabetes Control and Complications Trial, severe hypoglycemic events were 2- to 3-fold higher in the intervention arm (11) and 65% of patients in that arm experienced at least one severe hypoglycaemic event per year during the 7 years intervention (66). The risk of hypoglycemia is intimately linked with participating in PA for persons living with T1D (68–70). The fear of hypoglycaemia is a significant barrier to adopting a physically active lifestyle for persons with T1D (71, 72). While prolonged intervention and cohort studies provide insight into the long-term health benefits accrued with increased PA, studies of a single bout of exercise provide insight to the role of exercise on acute glucose control and the risk of hypoglycemia (73–75).

Exercise-induced hypoglycemia occurs secondary to a rapid increase in glucose uptake and insulin sensitivity (76–78) that persists for up to 48 h following exercise (76). As little as 30 min of moderate intensity PA increases the risk of nocturnal hypoglycemia by as much as 30% (79), while 75 min of moderate intensity PA triples the rate of nocturnal hypoglycemia (69). Several strategies exist to prevent exercise-induced hypoglycemia in patients with T1D. They include withholding pre-exercise insulin (80, 81), increasing carbohydrate intake during or following exercise (80, 82) and reducing basal or night time insulin (68). Unfortunately, as these strategies all result in transient hyperglycaemia (73), they compromise glycaemic control (68, 83), which can be an undesired consequence of exercise for patients who desire tight glycaemic control.

### Vigorous Intensity Physical Activity and Glucose Control in Persons With Type 1 Diabetes

Physical activities can be stratified into light, moderate and vigorous intensity activities (84, 85). Moderate intensity PA is defined as exercise that requires 3–6 metabolic equivalents (METS) of energy expenditure (equivalent to walking 4.0–6.8 km/h). Exercising at this intensity significantly increases glucose disposal, enhances insulin sensitivity and improves metabolic health outcomes (85–88). In individuals without T1D, insulin levels decrease at the onset of moderate-intensity PA to counter the enhanced glucose uptake and protect individuals from hypoglycemia (89). In individuals with T1D, insulin is supplied exogenously and does not decrease at the onset of exercise (89). The combination of insulin- and contraction-mediated glucose uptake significantly increase their risk for hypoglycemia during and after exercise (90). As moderate intensity PA is associated with health benefits and is perceived to be easy for most sedentary patients, it is the most commonly recommended intensity of PA by health care professionals and clinical guidelines (81, 82). However, this approach may exacerbate the fear of hypoglycaemia (71), as it is the intensity with which hypoglycemia is most likely to occur.

Similar to moderate intensity PA, exercising at >85% of maximal oxygen consumption (VO_2_max—i.e., vigorous intensity PA) significantly increases glucose disposal into skeletal muscle (89). In contrast to moderate-intensity PA however, vigorous intensity PA also causes a rapid and sustained increase in counter-regulatory hormones (catecholamines, glucagon, and cortisol) (14, 91, 92). This surge in hormones stimulates glucose output from the liver (89, 93) and partially reduces glucose uptake into skeletal muscle (94). This hormonal surge and accompanying increase in hepatic glucose output (89) causes mild, transient hyperglycaemia, especially in individuals with T1D (91, 92). Several acute, cross-over laboratory-based trials suggest that adding vigorous intensity intervals to standard moderate intensity exercise sessions (the typical type of exercise prescribed by diabetes educators) stabilizes blood glucose during and following exercise, thereby preventing hypoglycemia (73).

### Vigorous Intensity Exercise Can Prevent Hypoglycaemia?

Recent single-session laboratory-based studies have examined the potential role of adding high intensity intervals exercise sessions to combat the post-exercise hypoglycemic risk associated with moderate-intensity activity. A number of investigators have cleverly capitalized on this physiological response and prevention strategy over the past 10 years. Our group (73) and others (75) completed systematic reviews with a meta-analysis of studies of the acute blood glucose response to exercise (91, 92, 95–99) to determine the magnitude of this effect. We restricted analyses to studies that directly compared intermittent vigorous intensity exercise to continuous moderate intensity exercise on post-exercise glucose control during a single exercise session using a randomized cross-over trial design. Five of six studies found that adding bouts of vigorous intensity exercise lasting 4–15 s at 80–100% of VO_2_max with ~2 min of rest increased counter-regulatory hormones 2- to 4-fold above levels seen with moderate intensity exercise and reduced post-exercise hypoglycemia by 30–70%(91, 92, 96, 97, 99, 100). One study by Riddell et al. (14) found that adding vigorous intensity sprint intervals (15 s at 100% of VO_2_max) effectively normalized nocturnal blood glucose levels and decreased the time spent in hypoglycemia (≤ 3.9 mmol/L) by >70% (97). The overall effect of adding vigorous intensity exercise to a standard moderate intensity exercise session was a 38% reduction in post-exercise hypoglycemia. These data provide evidence that adding vigorous intensity PA to a standard moderate intensity exercise may prevent hypoglycemia acutely. However, no randomized controlled trial has ever determined if this approach would prevent hypoglycemia over the course of a longer exercise intervention. Therefore, the practical implications of this strategy remain in question.

## Controversy Surrounding Vigorous Intensity Physical Activity and Glucose Control in Type 1 Diabetes

In an attempt to translate these observations into a real-world context, our lab recently completed a series of studies examining the acute effects of adding vigorous intensity exercise to moderate intensity exercise sessions on hypoglycemia and glucose variability for the subsequent 24 h (101). In contrast to previous studies, we relied on running-based intervals (vs. cycling), administered the intervals in the late afternoon, when most people select to train, and did not rigorously control glucose levels, to reflect a persons daily variation in glucose. In studies of sedentary persons across multiple intensities of intervals (101) followed for a week during their training and in the lab following a session of vigorous intensity intervals (1 min at 90% of max; 2 min easy), we were unable to replicate these findings. Several other groups were unable to replicate the effects of vigorous intensity exercise on hypoglycemic risk seen in these earlier studies (98, 102, 103). These findings suggest that the practical application of adding vigorous intensity intervals to a moderate exercise session for predictable prevention of hypoglycemia remains uncertain.

In addition to these studies, recent work in Denmark suggests that the addition of high intensity exercise to a regular exercise training regimen may be hazardous among individuals who are hypoglycemia unaware (104, 105). This is a dangerous observation as reducing the physiological response to hypoglycemia is a major risk factor for severe hypoglycemic events. Taken together, there is growing evidence that vigorous intensity exercise may not be the key to improving metabolic control in persons with T1D and should be done progressively and consciously of its effects on counter-regulatory response to hypoglycemia.

### The Influence of Physical Activity on Glucose Variability in Persons With T1D

One of the most robust predictors of hypoglycemia in persons with diabetes is the degree of glucose variability (106–108). Glucose variability is defined as the magnitude of changes in glucose beyond what is usually or normally expected for an individual (81, 82). Identification of glucose variability relies on frequent, systematic glucose monitoring (109, 110), which can provide a sensitive, quantifiable measure of glycemic variance over a given time period. For example, while two individuals may display similar HbA1c levels, they may differ significantly on the frequency or magnitude of glucose dispersion from fasting levels, particularly in the post-prandial period (110, 111). Data from 3,100 episodes of hypoglycemia from 655 patients in the DCCT found that the risk of hypoglycemia increased in a dose-response manner with increasing glucose variability (106). With the widespread use of continuous glucose monitors in clinical management and research (112), more sensitive and robust measures of glycemic control and variability are available as outcomes for exercise studies (113–115). With the advances made with continuous glucose monitors, there are limitless opportunities to examine the influence of exercise on glucose variability (16), the role of glucose monitors in supporting safe and predictable adoption of a more active lifestyle and the long-term impact how achieving time in target range can improve the perceived benefits of exercise by persons living with T1D. To date, no study has ever examined the chronic effects of exercise training on glucose variability in persons with T1D. With the increasing use and availability of continuous glucose monitoring (112), there is a huge opportunity to study the impact of exercise at various doses on glucose variability, particularly when performed at different times of the day. A summary of what is currently known regarding vigorous intensity PA and health among persons with T1D and the major perceived gaps in the literature is provided in Table 2.

**Table 2:** Vigorous intensity intervals and hypoglycemia risk in persons with type 1 diabetes.

**Type of study**	**Physical activity knowledge**	**Gaps**
Carefully controlled laboratory Studies—Tight glucose control; mid-morning activity	Attenuates decline in glucose during exercise Reduced post-exercise decline in blood glucose	Influence of timing (morning, afternoon, evening) unclear. Little data on glucose variability
Laboratory-based studies, less control, mid afternoon	No influence on hypoglycemia risk or glucose variability	Minimal intensity needed to alter glucose response undetermined Unclear if gender or fitness influence the effect
Epidemiological or Clinical trial Data	None	No observational studies comparing time spent in different PA intensities on health No long term trials existing comparing vigorous to moderate intensity training on health outcomes

## Patient-Oriented Research Within the Field Physical Activity and Health Outcomes Among Persons Living With Type 1 Diabetes

With a dearth of evidence within nearly every sub-field of PA and T1D research, opportunities are limitless to launch clinically meaningful studies that are aligned with the priorities of the individuals living with T1D. Patient-oriented research is founded upon four main tenets of practice: (1) inclusive research processes; (2) collaborating respectfully with stakeholder populations; (3) recognizing the value of patient experiences with conditions and; (4) conducting research informed by the needs of the patients (116). As there are major gaps across all study designs in the field of PA and T1D, research should be encouraged to engage patients when addressing the gaps identified above (117). The Canadian Institutes of Health Research (CIHR), a major funding body of research in Canada, launched the Strategy for Patient-Oriented Research in 2010 to fund and facilitate research projects involving patients (118, 119). Patient-centered medicine, the provision of personalized medical care that accounts for the needs and preferences of individual patients (120) is ideal for this field as both patients and providers (121, 122) struggle with including PA in routine self-management plans. In an effort to make PA research more relevant to patients and providers, PA-based interventions for persons with T1D should include patient-reported outcomes and explore models of shared decision making for adopting a more active lifestyle.

Patient engagement in PA and T1D research programs is in its infancy, relative to other areas of endocrine research. The two main goals for engaging patients in PA-based research are to improve health outcomes during interventions and optimizing the patient experience of health care (123). In terms of research, CIHR defines patient engagement as “meaningful and active collaboration in governance, priority setting, conducting research and knowledge translation” (119). In the United Kingdom, the term “public involvement” is preferred and supported by the National Institutes of Health Research to describe this same concept [NIH (124) Public Involvement]. Engaging patients within PA and T1D research programs could facilitate the implementation and relevance of study findings, ease the recruitment process, improve validity/credibility of results and address disparities between funded research and end user needs (116, 124). All of these are major limitations to the research summarized above.

The first step in the process of patient engagement for PA-related research for persons living with T1D is to set priorities from patients, caregivers and providers. Several recent examples for persons living with type 1 and type 2 diabetes serve as models for future research programs. One example is a large multinational survey study published in 2013 collected data from 16,000 individuals with a significant connection to type 2 diabetes (i.e., patients, family members, and health care providers) (125). The survey queried these stakeholders regarding perceived gaps in diabetes health care and suggestions on improving care. The James Lind Alliance (JLA) is an initiative funded by the National Institutes of Health Research in the United Kingdom (126, 127). This group has developed an internationally recognized method of establishing patient priorities for treatment research and has worked on projects spanning over 100 different diseases (128). This process involves the distribution of an open-ended survey to the public regarding questions about treatments, and partners with stakeholders to prioritize the responses into a top ten list of research questions (129). This partnership approach has created research priorities for persons living with T1D (130) (Table 3), type 2 diabetes (131), hypertension, and childhood disability. The JLA model is an excellent starting point for determining the next steps for PA research with persons living with T1D as it will provide scientists with the topics more relevant and meaningful to patients.

**Table 3:** Patient, caregiver and provider priorities for research related to type 1 diabetes.

**James lind alliance top 10 research questions**	
1	Is it possible to constantly and accurately monitor blood sugar levels, in people with type 1 diabetes, with a discrete device (non-invasive or invasive)?
2	Is insulin pump therapy effective? (immediate v deferred pump, and comparing outcomes with multiple injections)
3	Is an artificial pancreas for type 1 diabetes (closed loop system) effective?
4	What are the characteristics of the best type 1 diabetes patient education programmes (from diagnosis to long term care) and do they improve outcomes?
5	What are the cognitive and psychological effects of living with type 1 diabetes?
6	How can awareness of and prevention of hypoglycaemia in type 1 diabetes be improved?
7	How tightly controlled do fluctuations in blood glucose levels need to be to reduce the risk of developing complications in people with type 1 diabetes?
8	Does treatment of type 1 diabetics by specialists (e.g., doctors, nurses, dieticians, podiatrists, ophthalmologists, and psychologists) trained in person-centred skills provide better blood glucose control, patient satisfaction and self-confidence in management of type 1 diabetes, compared to treatment by non-specialists with standard skills?
9	What makes self-management successful for some people with type 1 diabetes, and not others?
10	Which insulins are safest and have the fewest (long term) adverse effects?

### Examples of Patient Engagement in Research for Persons Living With T1D

In 2012, data from a piloted intervention became available that tested the effectiveness of an iPhone application named “bant” to increase the frequency of blood glucose testing in youth (132). The app was designed, developed and pilot tested in consultation with patients, parents and health care providers and was found to be moderately effective in increasing the number of blood glucose tests per day. Another eHealth intervention engaged young patients, parents, health care providers, and teachers in designing an online education program aimed at helping youth understand appropriate insulin adjustments to account for food intake (133). The youth patients were involved in designing the host website and address potential barriers to user-friendliness, and the collective steering group worked in unison to develop the content of the program (Kids In Control OF Food—KICk-OFF). To the best of our knowledge, similar patient-developed, exercise-focused applications have yet to be developed or tested for individuals living with T1D.

Perhaps one of the largest examples of patient engagement in T1D research is the D1 Now study in Ireland (134, 135). This project recruited a young adult panel as co-investigators who were involved in writing grants, organizing a large international workshop, writing plain English summaries in journal articles and promoting the study using social media outlets. One purpose of the consensus conference organized by the panel was to obtain consensus from a wide range of stakeholders regarding a core outcome set to be measured in a new complex intervention trial for improving blood glucose monitoring in T1D youth (135). The conference was attended by 110 individuals from seven different countries, including Canada. This process resulted in consensus on eight core outcomes to be included in the trial: (1) measures of diabetes-related stress; (2) diabetes-related quality of life; (3) number of severe hypoglycemic events; (4) self-management behaviors; (5) number of diabetic ketoacidosis events; (6) HbA1c; (7) level of clinic engagement and; (8) perceived level of diabetes control (135). The conference also included a “Hackathon” that paired computer programmers with diabetes stakeholders to create technological supports for T1D youth (134), where the winning pitch involved the use of motivational and informational content on an existing popular social media platform, Snapchat. These outcomes should be included in the design of future clinical trials and cohort studies of PA and health outcomes among persons living with T1D as they reflect the priorities of young people and could be harmonized for replication of findings with the D1 Now study group.

The James Lind Alliance published the results from their T1D partnership in 2012 (130). This partnership engaged 10 stakeholders in prioritizing the 1,259 initially submitted research questions to develop a top 10 list (Table 3). This list included but was not limited to questions pertaining to the artificial pancreas, prevention of hypoglycemia, insulin pump therapy and long-term effects of various insulin analogs. A secondary analysis compared this list to recently funded research projects and found that several of the research topics were being pursued, but many were also not (136). Areas of disagreement between patient priorities and funded research included health care cost-effectiveness of providing additional blood test strips to patients, disparities in regular diabetes care, development of alternative methods of insulin delivery, psychosocial health and the female reproductive health cycle. Although the JLA process is highly recognized, the T1D partnership was not without its limitations. Snow and colleagues examined the research questions that were submitted by stakeholders but excluded in early phases of data analysis (137). Incredibly, they found that having lived experience (patient or caregiver) of T1D increased the likelihood of having a research question rejected relative to questions provided by health care providers. This project also thematically analyzed the excluded questions and discovered four major themes of researchable questions that were not analyzed due to the inclusion criteria: (1) questions concerned with finding a cure for T1D; (2) questions concerned with the cause or possible prevention of T1D; (3) questions to further understand the mechanisms of the disease (i.e., blood glucose fluctuations, risk factors etc.) and; (4) questions concerned with practice and policy changes.

### Patient Reported Outcomes and Experiences

A starting point for working with patient partners in PA and health outcomes research for persons living with T1D, would be to ensure that future studies include patient reported outcome measures and patient reported experiences, as they pertain to PA. With regards to patient reported outcome measures, simple tools including quality of life, diabetes-related quality of life and measures related to self-management are standard in other clinical trials. As PA can be viewed as burdensome to patients, potentially increasing the challenging of achieving target glucose control, these measures become even more important for providers and caregivers.

The patient experience is a key element of quality of care and health systems improvement. The experience of a provider's confidence prescribing activity, ability to tailor management to meet the needs of individuals active lifestyle and ability to share in the decision making process around including PA in a person's self-management plan are examples of patient experiences that could limit engagement in regular daily PA. Our group intends to conduct an extensive scoping review of clinical trials published to date, to identify if any trials have been conducted that included patient-reported outcome measures, patient experiences or evidence of patient engagement in research related to PA. To the best of our knowledge, these experiences have yet to be captured within PA-based trials, therefore best practices for enhancing the experience of providing PA consultation to persons living with T1D remains unclear.

## Conclusion and Summary

**Figure 1:**
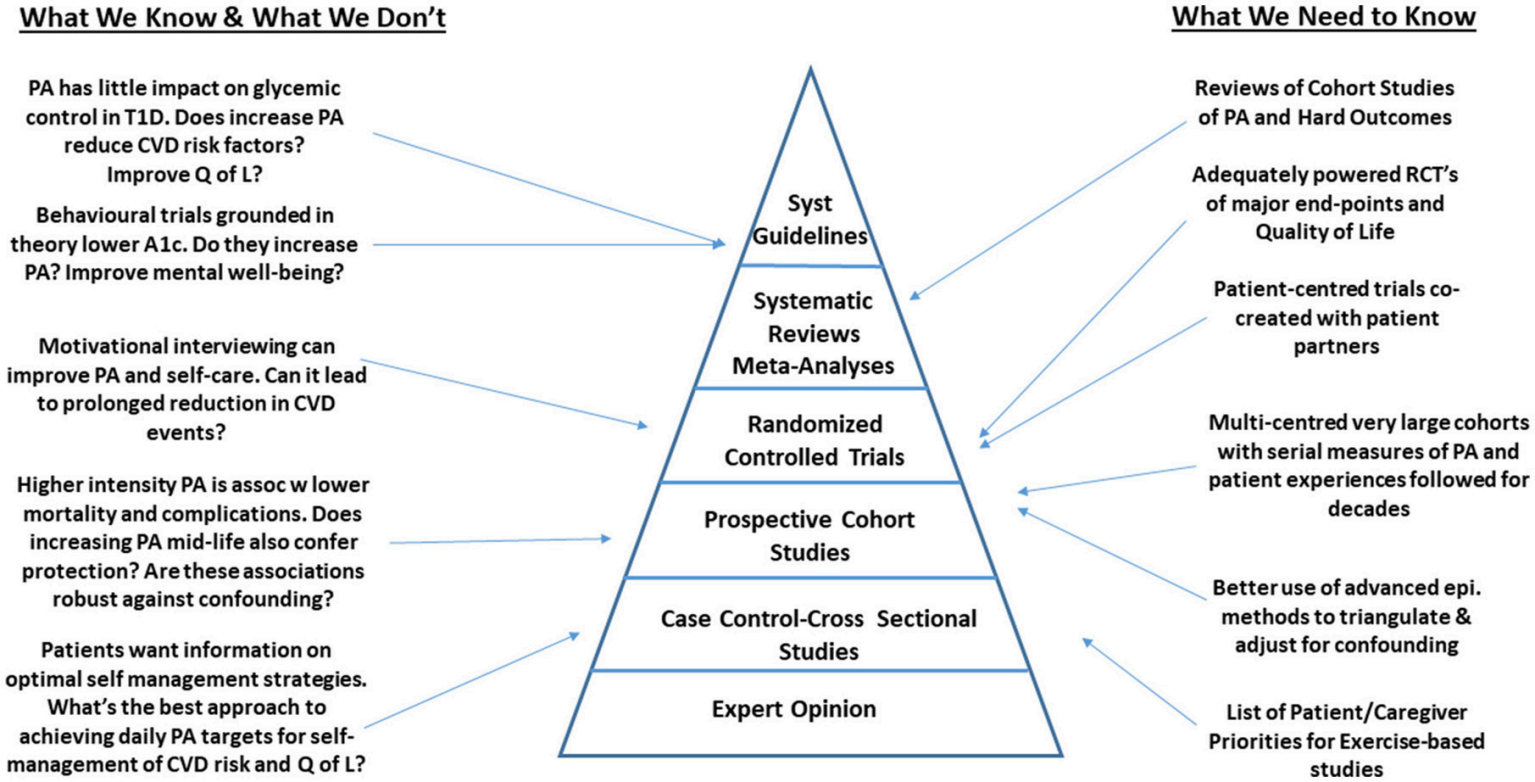
Gaps in the field of physical activity and type 1 diabetes research.

We recognize that the studies described about were not systematically selected and therefore are at risk of a selection bias. However, we attempted to focus on the most robust, representative and clinically relevant studies published to date, in an effort to identify gaps in the most rigorously designed studies to date. Despite this limitation, among the studies described, we identified that the area of PA and health among persons living with T1D is rife with gaps that limit the uptake of this work into clinical practice (Figure 1). Across the spectrum of study designs, there are multiple opportunities for scientists to make meaningful contributions to the field. As outlined in Figure 1, many of the gaps in epidemiological research that would inform guidelines, could be addressed through large scale, multi-center observational studies and clinical trials focused on hard health-related outcomes. These studies could be designed in a way that would facilitate uptake into clinical practice by including patient partners throughout the research process. Partnering with experts in patient engagement and patient reported outcome/experience measures will facilitate this process.

## Author Contributions

NK and JM designed the concept for the manuscript. NK, AM, and JM all contributed to writing and revising the manuscript. NK and AM reviewed abstracts and extracted data for the systematic review. All authors read and approved the final version.

### Conflict of Interest Statement

JM has received speaker fees from Medtronic Inc. The remaining authors declare that the research was conducted in the absence of any commercial or financial relationships that could be construed as a potential conflict of interest.

## References

[1] Berhan Y Waernbaum I Lind T Mollsten A Dahlquist G. Thirty years of prospective nationwide incidence of childhood type 1 diabetes: the accelerating increase by time tends to level off in Sweden. Diabetes (2011) 60:577–81. 10.2337/db10-0813 21270269 PMC3028358

[2] DIAMOND Project Group Incidence and trends of childhood Type 1 diabetes worldwide 1990-1999. Diabetes Med. (2006) 23:857–66. 10.1111/j.1464-5491.2006.01925.x 16911623

[3] Patterson CC Dahlquist GG Gyurus E Green A Soltesz G. Incidence trends for childhood type 1 diabetes in Europe during 1989-2003 and predicted new cases 2005-20: a multicentre prospective registration study. Lancet (2009) 373:2027–33. 10.1016/S0140-6736(09)60568-7 19481249

[4] Vehik K Dabelea D. The changing epidemiology of type 1 diabetes: why is it going through the roof? Diabetes Metab Res Rev. (2011) 27:3–13. 10.1002/dmrr.1141 21218503

[5] Silink M. Childhood diabetes: a global perspective. Horm Res. (2002) 57(Suppl. 1):1–5. 10.1159/000053304 11979014

[6] Dorman JS Laporte RE Kuller LH Cruickshanks KJ Orchard TJ Wagener DK. The Pittsburgh insulin-dependent diabetes mellitus (IDDM) morbidity and mortality study. Mortality results. Diabetes (1984) 33:271–6. 10.2337/diab.33.3.271 6698317

[7] Laing SP Swerdlow AJ Slater SD Botha JL Burden AC Waugh NR. The British Diabetic Association cohort study, I: all-cause mortality in patients with insulin-treated diabetes mellitus. Diabetes Med. (1999) 16:459–65. 10.1046/j.1464-5491.1999.00075.x 10391392

[8] Laing SP Swerdlow AJ Slater SD Burden AC Morris A Waugh NR. Mortality from heart disease in a cohort of 23,000 patients with insulin-treated diabetes. Diabetologia (2003) 46:760–5. 10.1007/s00125-003-1116-6 12774166

[9] Laing SP Swerdlow AJ Carpenter LM Slater SD Burden AC Botha JL. Mortality from cerebrovascular disease in a cohort of 23 000 patients with insulin-treated diabetes. Stroke (2003) 34:418–21. 10.1161/01.STR.0000053843.03997.35 12574553

[10] Dawson SI Willis J Florkowski CM Scott RS. All-cause mortality in insulin-treated diabetic patients: a 20-year follow-up. Diabetes Res Clin Pract. (2008) 80:e6–9. 10.1016/j.diabres.2007.10.034 18329123

[11] Diabetes Control and Complications Trial Research Group Nathan DM Genuth S Lachin J Cleary P Crofford O. The effect of intensive treatment of diabetes on the development and progression of long-term complications in insulin-dependent diabetes mellitus. N Engl J Med. (1993) 329:977–86. 10.1056/NEJM199309303291401 8366922

[12] Nathan DM Cleary PA Backlund JY Genuth SM Lachin JM Orchard TJ. Intensive diabetes treatment and cardiovascular disease in patients with type 1 diabetes. N Engl J Med. (2005) 353:2643–53. 10.1056/NEJMoa052187 16371630 PMC2637991

[13] Tamborlane WV. Triple jeopardy: nocturnal hypoglycemia after exercise in the young with diabetes. J Clin Endocrinol Metab. (2007) 92:815–6. 10.1210/jc.2007-0016 17341578

[14] Riddell MC Gallen IW Smart CE Taplin CE Adolfsson P Lumb AN. Exercise management in type 1 diabetes: a consensus statement. Lancet Diabetes Endocrinol. (2017) 5:377–90. 10.1016/S2213-8587(17)30014-1 28126459

[15] Gallen IW. Exercise for people with type 1 diabetes. Med Sport Sci. (2014) 60:141–53. 10.1159/000357344 25226809

[16] Houlder SK Yardley JE. Continuous glucose monitoring and exercise in type 1 diabetes: past, present and future. Biosensors (2018) 8:E73. 10.3390/bios8030073 30081478 PMC6165159

[17] Mottalib A Kasetty M Mar JY Elseaidy T Ashrafzadeh S Hamdy O. Weight management in patients with type 1 diabetes and obesity. Curr Diab Rep. (2017) 17:92. 10.1007/s11892-017-0918-8 28836234 PMC5569154

[18] Driscoll KA Corbin KD Maahs DM Pratley R Bishop FK Kahkoska A Advancing care for type, and obesity N, biopsychosocial aspects of weight management in type 1 diabetes: a review and next steps. Curr Diab Rep. (2017) 17:58 10.1007/s11892-017-0892-1 28660565 PMC6053070

[19] Codella R Terruzzi I Luzi L. Why should people with type 1 diabetes exercise regularly? Acta Diabetol. (2017) 54:615–30. 10.1007/s00592-017-0978-x 28289908

[20] Aune D Norat T Leitzmann M Tonstad S Vatten LJ. Physical activity and the risk of type 2 diabetes: a systematic review and dose-response meta-analysis. Eur J Epidemiol. (2015) 30:529–42. 10.1007/s10654-015-0056-z 26092138

[21] Samitz G Egger M Zwahlen M. Domains of physical activity and all-cause mortality: systematic review and dose-response meta-analysis of cohort studies. Int J Epidemiol. (2011) 40:1382–400. 10.1093/ije/dyr112 22039197

[22] Lear SA Hu W Rangarajan S Gasevic D Leong D Iqbal R. The effect of physical activity on mortality and cardiovascular disease in 130 000 people from 17 high-income, middle-income, and low-income countries: the PURE study. Lancet (2017) 390:2643–54. 10.1016/S0140-6736(17)31634-3 28943267

[23] Arem H Moore SC Patel A Hartge P Berrington de Gonzalez A Visvanathan K. Leisure time physical activity and mortality: a detailed pooled analysis of the dose-response relationship. JAMA Intern Med. (2015) 175:959–67. 10.1001/jamainternmed.2015.0533 25844730 PMC4451435

[24] O'Donovan G Lee IM Hamer M Stamatakis E. Association of “Weekend Warrior” and other leisure time physical activity patterns with risks for all-cause, cardiovascular disease, and cancer mortality. JAMA Intern Med. (2017) 177:335–42. 10.1001/jamainternmed.2016.8014 28097313

[25] Agoritsas T Merglen A Shah ND O'Donnell M Guyatt GH. Adjusted analyses in studies addressing therapy and harm: users' guides to the medical literature. JAMA (2017) 317:748–59. 10.1001/jama.2016.20029 28241362

[26] Hu Y Zong G Liu G Wang M Rosner B Pan A. Smoking cessation, weight change, type 2 diabetes, and mortality. N Engl J Med. (2018) 379:623–32. 10.1056/NEJMoa1803626 30110591 PMC6165582

[27] Sotos-Prieto M Bhupathiraju SN Mattei J Fung TT Li Y Pan A. Association of changes in diet quality with total and cause-specific mortality. N Engl J Med. (2017) 377:143–53. 10.1056/NEJMoa1613502 28700845 PMC5589446

[28] Chimen M Kennedy A Nirantharakumar K Pang TT Andrews R Narendran P. What are the health benefits of physical activity in type 1 diabetes mellitus? A literature review. Diabetologia (2012) 55:542–51. 10.1007/s00125-011-2403-2 22189486

[29] Campaigne BN Gilliam TB Spencer ML Lampman RM Schork MA. Effects of a physical activity program on metabolic control and cardiovascular fitness in children with insulin-dependent diabetes mellitus. Diabetes Care (1984) 7:57–62. 10.2337/diacare.7.1.57 6705666

[30] Dahl-Jorgensen K Meen HD Hanssen KF Aagenaes O. The effect of exercise on diabetic control and hemoglobin A1 (HbA1) in children. Acta Paediatr Scand Suppl. (1980) 283:53–6. 10.1111/j.1651-2227.1980.tb15313.x 6938115

[31] D'Hooge R Hellinckx T Van Laethem C Stegen S De Schepper J Van Aken S. Influence of combined aerobic and resistance training on metabolic control, cardiovascular fitness and quality of life in adolescents with type 1 diabetes: a randomized controlled trial. Clin Rehabil. (2011) 25:349–59. 10.1177/0269215510386254 21112904

[32] Durak EP Jovanovic-Peterson L Peterson CM. Randomized crossover study of effect of resistance training on glycemic control, muscular strength, and cholesterol in type I diabetic men. Diabetes Care (1990) 13:1039–43. 10.2337/diacare.13.10.1039 2209300

[33] Fuchsjager-Mayrl G Pleiner J Wiesinger GF Sieder AE Quittan M Nuhr MJ. Exercise training improves vascular endothelial function in patients with type 1 diabetes. Diabetes Care (2002) 25:1795–801. 10.2337/diacare.25.10.1795 12351480

[34] Laaksonen DE Atalay M Niskanen LK Mustonen J Sen CK Lakka TA. Aerobic exercise and the lipid profile in type 1 diabetic men: a randomized controlled trial. Med Sci Sports Exerc. (2000) 32:1541–8. 10.1097/00005768-200009000-00003 10994902

[35] Landt KW Campaigne BN James FW Sperling MA. Effects of exercise training on insulin sensitivity in adolescents with type I diabetes. Diabetes Care (1985) 8:461–5. 10.2337/diacare.8.5.461 4053932

[36] Lehmann R Kaplan V Bingisser R Bloch KE Spinas GA. Impact of physical activity on cardiovascular risk factors in IDDM. Diabetes Care (1997) 20:1603–11. 10.2337/diacare.20.10.1603 9314643

[37] Mosher PE Nash MS Perry AC LaPerriere AR Goldberg RB. Aerobic circuit exercise training: effect on adolescents with well-controlled insulin-dependent diabetes mellitus. Arch Phys Med Rehabil. (1998) 79:652–7. 10.1016/S0003-9993(98)90039-9 9630144

[38] Roberts L Jones TW Fournier PA. Exercise training and glycemic control in adolescents with poorly controlled type 1 diabetes mellitus. J Pediatr Endocrinol Metab. (2002) 15:621–7. 10.1515/JPEM.2002.15.5.621 12014521

[39] Salem MA Aboelasrar MA Elbarbary NS Elhilaly RA Refaat YM. Is exercise a therapeutic tool for improvement of cardiovascular risk factors in adolescents with type 1 diabetes mellitus? A randomised controlled trial. Diabetol Metab Syndr. (2010) 2:47. 10.1186/1758-5996-2-47 20618996 PMC3238209

[40] Tunar M Ozen S Goksen D Asar G Bediz CS Darcan S. The effects of Pilates on metabolic control and physical performance in adolescents with type 1 diabetes mellitus. J Diabetes Complications (2012) 26:348–51. 10.1016/j.jdiacomp.2012.04.006 22609217

[41] Wallberg-Henriksson H Gunnarsson R Rossner S Wahren J. Long-term physical training in female type 1 (insulin-dependent) diabetic patients: absence of significant effect on glycaemic control and lipoprotein levels. Diabetologia (1986) 29:53–7. 10.1007/BF02427281 3956895

[42] Wiesinger GF Pleiner J Quittan M Fuchsjager-Mayrl G Crevenna R Nuhr MJ. Health related quality of life in patients with long-standing insulin dependent (type 1) diabetes mellitus: benefits of regular physical training. Wien Klin Wochenschr. (2001) 113:670–5. 11603101

[43] Yki-Jarvinen H DeFronzo RA Koivisto VA. Normalization of insulin sensitivity in type I diabetic subjects by physical training during insulin pump therapy. Diabetes Care (1984) 7:520–7. 10.2337/diacare.7.6.520 6391876

[44] Zinman B Zuniga-Guajardo S Kelly D. Comparison of the acute and long-term effects of exercise on glucose control in type I diabetes. Diabetes Care (1984) 7:515–9. 10.2337/diacare.7.6.515 6439529

[45] Bohn B Herbst A Pfeifer M Krakow D Zimny S Kopp F. Impact of physical activity on glycemic control and prevalence of cardiovascular risk factors in adults with type 1 diabetes: a cross-sectional multicenter study of 18,028 patients. Diabetes Care (2015) 38:1536–43. 10.2337/dc15-0030 26015557

[46] Kriska AM LaPorte RE Patrick SL Kuller LH Orchard TJ. The association of physical activity and diabetic complications in individuals with insulin-dependent diabetes mellitus: the Epidemiology of Diabetes Complications Study–VII. J Clin Epidemiol. (1991) 44:1207–14. 10.1016/0895-4356(91)90153-Z 1941015

[47] Moy CS Songer TJ LaPorte RE Dorman JS Kriska AM Orchard TJ. Insulin-dependent diabetes mellitus, physical activity, and death. Am J Epidemiol. (1993) 137:74–81. 10.1093/oxfordjournals.aje.a116604 8434575

[48] LaPorte RE Dorman JS Tajima N Cruickshanks KJ Orchard TJ Cavender DE. Pittsburgh Insulin-Dependent Diabetes Mellitus Morbidity and Mortality Study: physical activity and diabetic complications. Pediatrics (1986) 78:1027–33. 3786027

[49] Tikkanen-Dolenc H Waden J Forsblom C Harjutsalo V Thorn LM Saraheimo M. Physical activity reduces risk of premature mortality in patients with type 1 diabetes with and without kidney disease. Diabetes Care (2017) 40:1727–32. 10.2337/dc17-0615 29038314

[50] Tikkanen-Dolenc H Waden J Forsblom C Harjutsalo V Thorn LM Saraheimo M. Frequent and intensive physical activity reduces risk of cardiovascular events in type 1 diabetes. Diabetologia (2017) 60:574–80. 10.1007/s00125-016-4189-8 28013340

[51] Waden J Tikkanen HK Forsblom C Harjutsalo V Thorn LM Saraheimo M. Leisure-time physical activity and development and progression of diabetic nephropathy in type 1 diabetes: the FinnDiane Study. Diabetologia (2015) 58:929–36. 10.1007/s00125-015-3499-6 25634228

[52] Tinsley LJ Kupelian V D'Eon SA Pober D Sun JK King GL. Association of glycemic control with reduced risk for large-vessel disease after more than 50 years of type 1 diabetes. J Clin Endocrinol Metab. (2017) 102:3704–11. 10.1210/jc.2017-00589 28973526 PMC5630245

[53] Wade KH Richmond RC Davey Smith G. Physical activity and longevity: how to move closer to causal inference. Br J Sports Med. (2018) 52:890–1. 10.1136/bjsports-2017-098995 29545236 PMC6047155

[54] Yardley JE Hay J Abou-Setta AM Marks SD McGavock J. A systematic review and meta-analysis of exercise interventions in adults with type 1 diabetes. Diabetes Res Clin Pract. (2014) 106:393–400. 10.1016/j.diabres.2014.09.038 25451913

[55] Ostman C Jewiss D King N Smart NA. Clinical outcomes to exercise training in type 1 diabetes: a systematic review and meta-analysis. Diabetes Res Clin Pract. (2018) 139:380–91. 10.1016/j.diabres.2017.11.036 29223408

[56] Quirk H Blake H Tennyson R Randell TL Glazebrook C. Physical activity interventions in children and young people with Type 1 diabetes mellitus: a systematic review with meta-analysis. Diabet Med. (2014) 31:1163–73. 10.1111/dme.12531 24965376 PMC4232875

[57] Umpierre D Ribeiro PA Kramer CK Leitao CB Zucatti AT Azevedo MJ. Physical activity advice only or structured exercise training and association with HbA1c levels in type 2 diabetes: a systematic review and meta-analysis. JAMA (2011) 305:1790–9. 10.1001/jama.2011.576 21540423

[58] Gusso S Pinto T Baldi JC Derraik GB Cutfield WS Hornung T Exercise training improves but does not normalize left ventricular systolic and diastolic function in adolescents with type 1 diabetes. Diabetes Care (2017) 40:1264–72. 10.2337/dc16-2347 28720592

[59] Kime N Pringle A Exercise physical activity in people with Type 1 diabetes: the importance of behaviour change. Diabetes Res Clin Pract. (2018) 138:282–3. 10.1016/j.diabres.2018.02.024 29481817

[60] Ayling K Brierley S Johnson B Heller S Eiser C. Efficacy of theory-based interventions for young people with type 1 diabetes: a systematic review and meta-analysis. Br J Health Psychol. (2015) 20:428–46. 10.1111/bjhp.12131 25557718

[61] Narendran P Jackson N Daley A Thompson D Stokes K Greenfield S. Exercise to preserve beta-cell function in recent-onset Type 1 diabetes mellitus (EXTOD) - a randomized controlled pilot trial. Diabet Med. (2017) 34:1521–31. 10.1111/dme.13439 28905421

[62] Mayer-Davis EJ Maahs DM Seid M Crandell J Bishop FK Driscoll KA. Efficacy of the flexible lifestyles empowering change intervention on metabolic and psychosocial outcomes in adolescents with type 1 diabetes (FLEX): a randomised controlled trial. Lancet Child Adolesc Health (2018) 2:635–46. 10.1016/S2352-4642(18)30208-6 30119757 PMC6260973

[63] Knowler WC Barrett-Connor E Fowler SE Hamman RF Lachin JM Walker EA Diabetes Prevention Program Research, Reduction in the incidence of type 2 diabetes with lifestyle intervention or metformin. N Engl J Med. (2002) 346:393–403. 10.1056/NEJMoa012512 11832527 PMC1370926

[64] Tuomilehto J Lindstrom J Eriksson JG Valle TT Hamalainen H Ilanne-Parikka P Finnish Diabetes Prevention Study, prevention of type 2 diabetes mellitus by changes in lifestyle among subjects with impaired glucose tolerance. N Engl J Med. (2001) 344:1343–50. 10.1056/NEJM200105033441801 11333990

[65] The Look AHEAD Research Group (2010). Long-term effects of a lifestyle intervention on weight and cardiovascular risk factors in individuals with type 2 diabetes mellitus: four-year results of the Look AHEAD trial. Arch Intern Med. 170:1566–75. 10.1001/archinternmed.2010.334 20876408 PMC3084497

[66] Hypoglycemia in the diabetes control and complications trial The Diabetes Control and Complications Trial Research Group. Diabetes (1997) 46:271–86. 10.2337/diab.46.2.271 9000705

[67] Cryer PE. Mechanisms of hypoglycemia-associated autonomic failure in diabetes. N Engl J Med. (2013) 369:362–72. 10.1056/NEJMra1215228 23883381

[68] Tsalikian E Kollman C Tamborlane WB Beck RW Fiallo-Scharer R Fox L. Prevention of hypoglycemia during exercise in children with type 1 diabetes by suspending basal insulin. Diabetes Care (2006) 29:2200–4. 10.2337/dc06-0495 17003293 PMC1584283

[69] Tsalikian E Mauras N Beck RW Tamborlane WV Janz KF Chase HP. Impact of exercise on overnight glycemic control in children with type 1 diabetes mellitus. J Pediatr. (2005) 147:528–34. 10.1016/j.jpeds.2005.04.065 16227041 PMC2258153

[70] Galassetti P Riddell MC. Exercise and type 1 diabetes (T1DM). Comprehen Physiol. (2013) 3:1309–36. 10.1002/cphy.c110040 23897688

[71] Brazeau AS Rabasa-Lhoret R Strychar I Mircescu H. Barriers to physical activity among patients with type 1 diabetes. Diabetes Care (2008) 31:2108–9. 10.2337/dc08-0720 18689694 PMC2571055

[72] Lawton J Waugh N Barnard KD Noyes K Harden J Stephen J. Challenges of optimizing glycaemic control in children with Type 1 diabetes: a qualitative study of parents' experiences and views. Diabetes Med. (2014) 32:1063–70. 10.1111/dme.12660 25472898

[73] Yardley J Mollard R MacIntosh A MacMillan F Wicklow B Berard L. Vigorous intensity exercise for glycemic control in patients with type 1 diabetes. Can J Diabetes (2013) 37:427–32. 10.1016/j.jcjd.2013.08.269 24321725

[74] Yardley JE Sigal RJ. Exercise strategies for hypoglycemia prevention in individuals with type 1 diabetes. Diabetes Spectr. (2015) 28:32–8. 10.2337/diaspect.28.1.32 25717276 PMC4334090

[75] Garcia-Garcia F Kumareswaran K Hovorka R Hernando ME. Quantifying the acute changes in glucose with exercise in type 1 diabetes: a systematic review and meta-analysis. Sports Med. (2015) 45:587–99. 10.1007/s40279-015-0302-2 25616852

[76] Mikines KJ Sonne B Farrell PA Tronier B Galbo H. Effect of physical exercise on sensitivity and responsiveness to insulin in humans. Am J Physiol. (1988) 254:E248–59. 10.1152/ajpendo.1988.254.3.E248 3126668

[77] Richter EA Mikines KJ Galbo H Kiens B. Effect of exercise on insulin action in human skeletal muscle. J Appl Physiol. (1989) 66:876–85. 10.1152/jappl.1989.66.2.876 2496078

[78] Perseghin G Price TB Petersen KF Roden M Cline GW Gerow K. Increased glucose transport-phosphorylation and muscle glycogen synthesis after exercise training in insulin-resistant subjects. N Engl J Med. (1996) 335:1357–62. 10.1056/NEJM199610313351804 8857019

[79] Metcalf KM Singhvi A Tsalikian E Tansey MJ Zimmerman MB Esliger DW. Effects of moderate-to-vigorous intensity physical activity on overnight and next-day hypoglycemia in active adolescents with type 1 diabetes. Diabetes Care (2014) 37:1272–8. 10.2337/dc13-1973 24574352 PMC3994939

[80] Riddell MC Iscoe KE. Physical activity, sport, and pediatric diabetes. Pediatr Diabetes (2006) 7:60–70. 10.1111/j.1399-543X.2006.00146.x 16489976

[81] Canadian Diabetes Association Clinical Guidelines Expert Commmittee Canadian Diabetes Association 2013 clinical practice guidelines for the prevention and management of diabetes in canada. Can J Diabetes (2013) 37(Suppl. 1) S1–3. 10.1016/j.jcjd.2013.01.00 24070926

[82] King M Smith G Bartlett A. Treatments of homosexuality in Britain since the 1950s–an oral history: the experience of professionals. BMJ (2004) 328:427. 10.1136/bmj.37984.442419.EE 14751920 PMC344257

[83] Riddell MC Milliken J. Preventing exercise-induced hypoglycemia in type 1 diabetes using real-time continuous glucose monitoring and a new carbohydrate intake algorithm: an observational field study. Diabetes Technol Ther. (2011) 13:819–25. 10.1089/dia.2011.0052 21599515

[84] Comte M Hobin E Majumdar SR Plotnikoff RC Ball GD McGavock J. Patterns of weekday and weekend physical activity in youth in 2 Canadian provinces. Appl Physiol Nutr Metab. (2013) 38:115–9. 10.1139/apnm-2012-0100 23438220

[85] Hay J Maximova K Durksen A Carson V Rinaldi RL Torrance B. Physical activity intensity and cardiometabolic risk in youth. Arch Pediatr Adolesc Med. (2012) 166:1022–9. 10.1001/archpediatrics.2012.1028 22965682

[86] Goodyear LJ Kahn BB. Exercise, glucose transport, and insulin sensitivity. Annu Rev Med. (1998) 49:235–61. 10.1146/annurev.med.49.1.235 9509261

[87] Church T. Exercise in obesity, metabolic syndrome, and diabetes. Prog Cardiovasc Dis. (2011) 53:412–8. 10.1016/j.pcad.2011.03.013 21545927

[88] Sigal RJ Kenny GP Boule NG Wells GA Prud'homme D Fortier M Effects of aerobic training, resistance training, or both on glycemic control in type 2 diabetes: a randomized trial. Ann Intern Med. (2007) 147:357–69. 10.7326/0003-4819-147-6-200709180-00005 17876019

[89] Marliss EB Vranic M. Intense exercise has unique effects on both insulin release and its roles in glucoregulation: implications for diabetes. Diabetes (2002) 51(Suppl. 1):S271–83. 10.2337/diabetes.51.2007.S271 11815492

[90] Chu L Hamilton J Riddell MC. Clinical management of the physically active patient with type 1 diabetes. Phys Sports Med. (2011) 39:64–77. 10.3810/psm.2011.05.1896 21673486

[91] Bussau VA Ferreira LD Jones TW Fournier PA. The 10-s maximal sprint: a novel approach to counter an exercise-mediated fall in glycemia in individuals with type 1 diabetes. Diabetes Care (2006) 29:601–6. 10.2337/diacare.29.03.06.dc05-1764 16505513

[92] Bussau VA Ferreira LD Jones TW Fournier PA. A 10-s sprint performed prior to moderate-intensity exercise prevents early post-exercise fall in glycaemia in individuals with type 1 diabetes. Diabetologia (2007) 50:1815–8. 10.1007/s00125-007-0727-8 17583795

[93] Guelfi KJ Jones TW Fournier PA. New insights into managing the risk of hypoglycaemia associated with intermittent high-intensity exercise in individuals with type 1 diabetes mellitus: implications for existing guidelines. Sports Med. (2007) 37:937–46. 10.2165/00007256-200737110-00002 17953465

[94] Fahey AJ Paramalingam N Davey RJ Davis EA Jones TW Fournier PA. The effect of a short sprint on postexercise whole-body glucose production and utilization rates in individuals with type 1 diabetes mellitus. J Clin Endocrinol Metab. (2012) 97:4193–200. 10.1210/jc.2012-1604 22962428

[95] Guelfi KJ Ratnam N Smythe GA Jones TW Fournier PA. Effect of intermittent high-intensity compared with continuous moderate exercise on glucose production and utilization in individuals with type 1 diabetes. Am J Physiol Endocrinol Metab. (2007) 292:E865–70. 10.1152/ajpendo.00533.2006 17339500

[96] Guelfi KJ Jones TW Fournier PA. The decline in blood glucose levels is less with intermittent high-intensity compared with moderate exercise in individuals with type 1 diabetes. Diabetes Care (2005) 28:1289–94. 10.2337/diacare.28.6.1289 15920041

[97] Iscoe KE Riddell MC. Continuous moderate-intensity exercise with or without intermittent high-intensity work: effects on acute and late glycaemia in athletes with Type 1 diabetes mellitus. Diabetes Med. (2011) 28:824–32. 10.1111/j.1464-5491.2011.03274.x 21388440

[98] Maran A Pavan P Bonsembiante B Brugin E Ermolao A Avogaro A. Continuous glucose monitoring reveals delayed nocturnal hypoglycemia after intermittent high-intensity exercise in nontrained patients with type 1 diabetes. Diabetes Technol Ther. (2010) 12:763–8. 10.1089/dia.2010.0038 20807120

[99] Hubinger A Ridderskamp I Lehmann E Gries FA. Metabolic response to different forms of physical exercise in type I diabetics and the duration of the glucose lowering effect. Eur J Clin Invest. (1985) 15:197–203. 10.1111/j.1365-2362.1985.tb00168.x 2864257

[100] Guelfi KJ Jones TW Fournier PA. Intermittent high-intensity exercise does not increase the risk of early postexercise hypoglycemia in individuals with type 1 diabetes. Diabetes Care (2005) 28:416–8. 10.2337/diacare.28.2.416 15677802

[101] Rempel M MacIntosh AC Hay JL Bouchard D Cornish S Marks SD Vigorous intensity intervals and hypoglycemia in type 1 diabetes: a randomized cross over trial. Sci Rep. (2018) 8:15879 10.1038/s41598-018-34342-6 30367116 PMC6203731

[102] Moser O Tschakert G Mueller A Groeschl W Pieber TR Obermayer-Pietsch B. Effects of high-intensity interval exercise versus moderate continuous exercise on glucose homeostasis and hormone response in patients with type 1 diabetes mellitus using novel ultra-long-acting insulin. PLoS ONE (2015) 10:e0136489. 10.1371/journal.pone.0136489 26317981 PMC4552855

[103] Campbell MD West DJ Bain SC Kingsley MI Foley P Kilduff L. Simulated games activity vs continuous running exercise: a novel comparison of the glycemic and metabolic responses in T1DM patients. Scand J Med Sci Sports (2015) 25:216–22. 10.1111/sms.12192 24593125

[104] Wiegers EC Rooijackers HM Tack CJ Groenewoud H Heerschap A de Galan BE. Effect of exercise-induced lactate elevation on brain lactate levels during hypoglycemia in patients with type 1 diabetes and impaired awareness of hypoglycemia. Diabetes (2017) 66:3105–10. 10.2337/db17-0794 28935628

[105] Rooijackers HM Wiegers EC van der Graaf M Thijssen DH Kessels PC Tack CJ. A single bout of high-intensity interval training reduces awareness of subsequent hypoglycemia in patients with type 1 diabetes. Diabetes (2017) 66:1990–8. 10.2337/db16-1535 28420673

[106] Kilpatrick ES Rigby AS Goode K Atkin SL. Relating mean blood glucose and glucose variability to the risk of multiple episodes of hypoglycaemia in type 1 diabetes. Diabetologia (2007) 50:2553–61. 10.1007/s00125-007-0820-z 17882397

[107] Murata GH Hoffman RM Shah JH Wendel CS Duckworth WC. A probabilistic model for predicting hypoglycemia in type 2 diabetes mellitus: the Diabetes Outcomes in Veterans Study (DOVES). Arch Intern Med. (2004) 164:1445–50. 10.1001/archinte.164.13.1445 15249354

[108] Cox DJ Kovatchev BP Julian DM Gonder-Frederick LA Polonsky WH Schlundt DG. Frequency of severe hypoglycemia in insulin-dependent diabetes mellitus can be predicted from self-monitoring blood glucose data. J Clin Endocrinol Metab. (1994) 79:1659–62. 7989471 10.1210/jcem.79.6.7989471

[109] Wentholt IM Kulik W Michels RP Hoekstra JB DeVries JH. Glucose fluctuations and activation of oxidative stress in patients with type 1 diabetes. Diabetologia (2008) 51:183–90. 10.1007/s00125-007-0842-6 17994218

[110] DeVries JH. Glucose variability: where it is important and how to measure it. Diabetes (2013) 62:1405–8. 10.2337/db12-1610 23613566 PMC3636643

[111] Siegelaar SE Holleman F Hoekstra JB DeVries JH. Glucose variability; does it matter? Endocr Rev. (2010) 31:171–82. 10.1210/er.2009-0021 19966012

[112] Bergenstal RM. Continuous glucose monitoring: transforming diabetes management step by step. Lancet (2018) 391:1334–6. 10.1016/S0140-6736(18)30290-3 29459022

[113] Wolpert HA. Continuous glucose monitoring–coming of age. N Engl J Med. (2010) 363:383–4. 10.1056/NEJMe1006098 20587584

[114] Tamborlane WV Beck RW Bode BW Buckingham B Chase HP Clemons R. Continuous glucose monitoring and intensive treatment of type 1 diabetes. N Engl J Med. (2008) 359:1464–76. 10.1056/NEJMoa0805017 18779236

[115] Danne T Nimri R Battelino T Bergenstal RM Close KL DeVries JH. International consensus on use of continuous glucose monitoring. Diabetes Care (2017) 40:1631–40. 10.2337/dc17-1600 29162583 PMC6467165

[116] Curran JA Bishop A Chorney J MacEachern L Mackay R. Partnering with parents to advance child health research. Healthc Manage Forum (2018) 31:45–50. 10.1177/0840470417744568 29400092

[117] Concato J. Study design and “evidence” in patient-oriented research. Am J Respir Crit Care Med. (2013) 187:1167–72. 10.1164/rccm.201303-0521OE 23725613

[118] Mallidou AA Frisch N Doyle-Waters MM MacLeod LP Ward J Atherton P. Patient-Oriented Research Competencies in Health (PORCH) for patients, healthcare providers, decision-makers and researchers: protocol of a scoping review. Syst Rev. (2018) 7:101. 10.1186/s13643-018-0762-1 30025543 PMC6053801

[119] CIFH Research Strategy for Patient Oriented Rsearch (2018).

[120] Sacristan JA. Patient-centered medicine and patient-oriented research: improving health outcomes for individual patients. BMC Med Inform Decis Mak. (2013) 13:6. 10.1186/1472-6947-13-6 23294526 PMC3575265

[121] O'Brien MW Shields CA Oh PI Fowles JR. Health care provider confidence and exercise prescription practices of Exercise is Medicine Canada workshop attendees. Appl Physiol Nutr Metab. (2017) 42:384–90. 10.1139/apnm-2016-0413 28177736

[122] Shields CA Fowles JR Dunbar P Barron B McQuaid S Dillman CJ Increasing diabetes educators' confidence in physical activity and exercise counselling: the effectiveness of the “physical activity and exercise toolkit” training intervention. Can J Diabetes (2013) 37:381–7. 10.1016/j.jcjd.2013.08.265 24321718

[123] Higgins T Larson E Schnall R. Unraveling the meaning of patient engagement: a concept analysis. Pat Educ Counsel. (2017) 100:30–6. 10.1016/j.pec.2016.09.002 27665500

[124] Manafo E Petermann L Mason-Lai P Vandall-Walker V Patient engagement in Canada: a scoping review of the 'how' and 'what' of patient engagement in health research. Health Res Policy Syst. (2018) 16:5 10.1186/s12961-018-0282-4 29415734 PMC5804082

[125] Peyrot M Burns KK Davies M Forbes A Hermanns N Holt R. Diabetes Attitudes Wishes and Needs 2 (DAWN2): a multinational, multi-stakeholder study of psychosocial issues in diabetes and person-centred diabetes care. Diabetes Res Clin Pract. (2013) 99:174–84. 10.1016/j.diabres.2012.11.016 23273515

[126] Petit-Zeman S Firkins L Scadding JW. The James lind alliance: tackling research mismatches. Lancet (2010) 376:667–9. 10.1016/S0140-6736(10)60712-X 20801389

[127] Partridge N Scadding J. The James Lind alliance: patients and clinicians should jointly identify their priorities for clinical trials. Lancet (2004) 364:1923–4. 10.1016/S0140-6736(04)17494-1 15566996

[128] The James Lind Alliance,. The PSPs. (2018). Available online at: http://www.jla.nihr.ac.uk/priority-setting-partnerships/ (Accessed August 24, 2018).

[129] National Institute for Health Research,. The James Lind Alliance Guidebook. (2018). Available online at: www.jla.nihr.ac.uk

[130] Gadsby R Snow R Daly AC Crowe S Matyka K Hall B. Setting research priorities for Type 1 diabetes. Diabet Med. (2012) 29:1321–6. 10.1111/j.1464-5491.2012.03755.x 22823450

[131] Finer S Robb P Cowan K Daly A Robertson E Farmer A. Top ten research priorities for type 2 diabetes: results from the Diabetes UK-James Lind Alliance Priority Setting Partnership. Lancet Diabetes Endocrinol. (2017) 5:935–6. 10.1016/S2213-8587(17)30324-8 29092776

[132] Cafazzo JA Casselman M Hamming N Katzman DK Palmert MR. Design of an mHealth app for the self-management of adolescent type 1 diabetes: a pilot study. J Med Internet Res. (2012) 14:e70. 10.2196/jmir.2058 22564332 PMC3799540

[133] Price KJ Wales J Eiser C Knowles J Heller S Freeman J. Does an intensive self-management structured education course improve outcomes for children and young people with type 1 diabetes? The Kids In Control OF Food (KICk-OFF) cluster-randomised controlled trial protocol. BMJ Open (2013) 3:e002429. 10.1136/bmjopen-2012-002429 23355675 PMC3563116

[134] O'Hara MC Hynes L O'Donnell M Keighron C Allen G Caulfield A. Strength in Numbers: an international consensus conference to develop a novel approach to care delivery for young adults with type 1 diabetes, the D1 Now Study. Res Involv Engagem. (2017) 3:25. 10.1186/s40900-017-0076-9 29214056 PMC5713095

[135] Byrne M O'Connell A Egan AM Dinneen SF Hynes L O'Hara MC. A core outcomes set for clinical trials of interventions for young adults with type 1 diabetes: an international, multi-perspective Delphi consensus study. Trials (2017) 18:602. 10.1186/s13063-017-2364-y 29258565 PMC5735534

[136] Boddy K Cowan K Gibson A Britten N. Does funded research reflect the priorities of people living with type 1 diabetes? A secondary analysis of research questions. BMJ Open (2017) 7:e016540. 10.1136/bmjopen-2017-016540 28963289 PMC5623562

[137] Snow R Crocker JC Crowe S. Missed opportunities for impact in patient and carer involvement: a mixed methods case study of research priority setting. Res Involv Engagem. (2015) 1:7. 10.1186/s40900-015-0007-6 29062496 PMC5611607

